# Oxytocin-enhanced motivational interviewing group therapy for methamphetamine use disorder in men who have sex with men: study protocol for a randomized controlled trial

**DOI:** 10.1186/s13063-019-3225-7

**Published:** 2019-02-21

**Authors:** Christopher S. Stauffer, Jenna M. Moschetto, Scott M. McKernan, Elaine Hsiang, Brian Borsari, Joshua D. Woolley

**Affiliations:** 1 University of California, San Francisco, San Francisco VA Medical Center, San Francisco, CA USA; 2 Palo Alto University, Palo Alto, CA USA; 3 University of California, San Francisco, CA USA; 4 University of California, San Francisco School of Medicine, San Francisco, CA USA

**Keywords:** Methamphetamine use disorder, Meth, Stimulant, Oxytocin, Men who have sex with men, Motivational interviewing, Group therapy, Addiction, Drug-assisted psychotherapy

## Abstract

**Background:**

The prevalence of methamphetamine use disorder (MUD) in the United States has risen dramatically in the past four decades and is concentrated in populations such as men who have sex with men (MSM). Despite the public health consequences of MUD, there are no FDA-approved psychopharmacological treatments. Psychosocial treatment alone has been shown to reduce methamphetamine use, but high attrition rates limit treatment efficacy. Promising findings from animal models of MUD using exogenous oxytocin, a social neuropeptide, have set the stage for translational work. Along with unique anti-addiction effects, oxytocin holds a primary role in enhancing social salience and modulating stress. In humans, oxytocin administration, combined with evidence-based psychosocial interventions, may act synergistically to improve addiction treatment outcomes and improve retention rates in current MUD treatment.

**Methods/design:**

We are conducting a randomized, double-blind, placebo-controlled trial of oxytocin-enhanced motivational interviewing group therapy (MIGT). Oxytocin or placebo 40 IU is administered intranasally in conjunction with six, weekly MIGT sessions. We will recruit 50 MSM, initiating treatment for MUD from specialized community health programs in San Francisco, CA, USA. Individuals will be randomized (1:1) to receive six, weekly sessions of MIGT with or without oxytocin. Our primary outcome is session attendance. Other outcomes of interest include: measures of group cohesion, anxiety, psychophysiology, and stimulant craving and use.

**Discussion:**

This will be the first study of oxytocin’s effects in humans with MUD. Findings from this novel protocol will attempt to bridge existing animal data with the need for innovative clinical treatments for MUD, inform the growing field of pharmacologically-enhanced psychotherapy, and help to elucidate mechanisms behind oxytocin’s potential anti-addiction effects.

**Trial registration:**

ClinicalTrials.gov, ID: NCT02881177. Registered on 26 August 2016.

**Electronic supplementary material:**

The online version of this article (10.1186/s13063-019-3225-7) contains supplementary material, which is available to authorized users.

## Background

Methamphetamine is a potent psychostimulant with high abuse potential [1]. Methamphetamine and amphetamine-type stimulants are second only to cannabis as the most frequently used illicit substance worldwide [2], and, according to the 2017 Global Drug Survey [3], methamphetamine was the illicit substance most likely to lead to emergency medical treatment seeking. High healthcare utilization contributes to the multifactorial economic burden of methamphetamine, which is estimated to be upwards of US$23.4 billion in the United States (US) alone (Rand Corporation, 2008). The prevalence of methamphetamine use disorder (MUD) in the US has risen dramatically in the past four decades, with high concentrations among men who have sex with men (MSM) [4–6]. Despite the personal and societal consequences of methamphetamine use, no pharmacological agent currently has an FDA-designated indication to treat MUD [7, 8].

Psychosocial treatment alone has been shown to reduce methamphetamine use and rates of relapse, with a longer duration of treatment producing better results [9–13]. However, methamphetamine users exhibit high attrition rates of 60% or more, which limits the effectiveness of treatment and leads to missing data and validity issues in clinical research contexts [14]. This complicates efforts to identify pharmacotherapy candidates or to improve evidence-based psychosocial treatments for MUD [8, 15]. Methamphetamine is associated with impairments in social cognition and theory of mind, including a hypersensitivity to social threat, which likely contribute to reduced treatment adherence and retention [16–19]. Social rehabilitation, often in the form of group and family therapy, is a prominent component of gold-standard psychosocial treatments for MUD [20, 21]. Even in animal models of methamphetamine addiction, socially housed—versus individually housed—rats were protected against escalating methamphetamine self-administration [22]. Given the social complexities and challenges surrounding MUD clinical treatment and research, an adjunctive pharmacotherapy that might enhance social cognition and boost engagement and retention in effective psychosocial treatment could greatly improve outcomes [23].

Oxytocin, a hypothalamic peptide that plays a critical role in mammalian attachment and affiliative behavior [24–26], administered exogenously, is a promising candidate as an adjunctive addiction treatment [27, 28]. Oxytocin has unique anti-addiction effects [27, 29] along with social salience [30] and stress-tempering properties [31–33], which together may shift attentional bias toward positive social reward at the expense of conditioned drug-related reward [28, 34]. Oxytocin is a large, hydrophilic molecule, so does not readily cross the blood-brain barrier after peripheral administration. The intranasal route is thus far the most efficient way to deliver oxytocin to the human central nervous system [35, 36] and is extremely safe and tolerable [37]. What is more, there are no discernable subjective effects (i.e., research participants cannot typically guess whether they have received oxytocin versus placebo), which allows the use of an inactive placebo [37].

Methamphetamine self-administration in rats has been shown to dysregulate the oxytocin system [38–40], and some evidence for this exists in humans [41]. In animal models of methamphetamine addiction, oxytocin administration reduces the acquisition and increases extinction of compulsive methamphetamine self-administration and reduces stress-primed and drug-primed reinstatement of methamphetamine-seeking behavior [27, 42–47]. In addition, oxytocin administration attenuates methamphetamine tolerance and withdrawal, methamphetamine-induced stereotyped movements, methamphetamine-induced conditioned place preference, and reduces methamphetamine-induced c-Fos expression in areas of the basal ganglia implicated in addiction [44, 48–51]. In humans, oxytocin has demonstrated some efficacy in early trials with alcohol, cannabis, opioid, and cocaine users [52, 53]. To our knowledge, this will be the first study to investigate the effects of intranasally administered oxytocin on the symptoms of MUD in humans.

Oxytocin administration in combination with evidence-based group psychotherapy—oxytocin-enhanced motivational interviewing group therapy (OE-MIGT)—may synergistically enhance treatment outcomes and address the notoriously high drop-out rates evident in MUD populations. We aim to investigate the effectiveness of intranasally administered oxytocin on MIGT treatment engagement. Specifically, we hypothesize that oxytocin will increase attendance at treatment sessions. In addition, we will estimate effect sizes and assess procedures used to test oxytocin’s effects on group cohesion, anxiety, psychophysiological stress reactivity, and stimulant craving and use for future trials. This novel translational protocol attempts to bridge ample animal data with the need for innovative clinical treatments for MUD.

## Methods/design

### Study design

This is a randomized, double-blind, placebo-controlled study of OE-MIGT. Specifically, intranasally administered oxytocin or placebo will be administered in conjunction with each of six, weekly, 90-min MIGT sessions.

### Motivational interviewing group therapy (MIGT)

Motivational interviewing (MI) is defined as a “collaborative conversation style for strengthening a person’s own motivation and commitment to change” a behavior [54], be it increasing engagement in treatment or discontinuing or cutting down methamphetamine use. MI is client-centered; as a result, the client generates any and all treatment goals and the personalized plans for attaining them. When conducting MI in group settings, co-facilitators use MI-consistent techniques (open-ended questions, affirmations, reflections, and summarization) in the context of the spirit of MI (one of collaboration, compassion, acceptance, and evocation) in order to foster healthy interactions among members and co-facilitators that are focused on exploring healthy behavior change. MIGT co-facilitators aim to guide members through four general group phases: (1) Engagement – enlist members to “open up” by involving them in meaningful relationships based in understanding and acceptance, (2) Exploring Perspectives – collaboratively, and strategically, narrow the focus from cohesion-building to members’ perspectives about their current situations, (3) Broadening Perspectives – elicit perspectives and ideas about change, motivation to change, and hopes for success, and (4) Moving Into Action – develop specific plans for *how to* implement change [55]. In this way, MIGT can improve personal recognition of ambivalence, support autonomy, increase motivation and commitment to change, and improve treatment engagement and participation [55].

The MIGT cohorts in this study will consist of two co-facilitators, one will be a licensed psychiatrist and the other will be a mental health trainee (PhD, PsyD, or psychiatry resident) along with four to six group members. All MIGT sessions will be conducted at the Alliance Health Project in San Francisco, CA, USA. A semi-structured, closed-admission, time-limited (six sessions) OE-MIGT manual for methamphetamine users was developed for this study using guidance from Wagner and Ingersoll (2013) [55]. Included are four brief psychoeducational components for optional use during the first four sessions: stages of change, ambivalence and a decisional balance chart for methamphetamine use, attachment theory and social support, and managing stress. These topics aid in the Engagement and Exploring Perspectives phases of the group, are designed to maximize the effects of intranasally administered oxytocin on MIGT for MUD, and correspond to the specific objectives of this research study. The final two sessions are left unstructured in order to allow more time for Broadening Perspectives and Moving Into Action. Adherence raters will assess session transcripts using motivational interviewing treatment integrity (MITI) 4.2; a coding system designed to measure group facilitators’ adherence to MI [56]. Consistent with previous work by the research team, a fidelity checklist reflecting the content of the session will be developed in order to determine whether the intervention was delivered as intended. Prior to participant enrollment, facilitators will receive training from an MI expert to ensure competency [57, 58].

### Power analysis

A sample size of 25 per treatment condition will provide us with > 99% power to detect an effect size comparable to the large between-condition effect size (*d* = 1.44) observed for attendance from our preliminary 3-week trial in cocaine users [53] with a two-tailed alpha = 0.05. In addition, this sample size will provide 80% power to detect an effect size of 0.81 or greater.

### Setting and study population

Previous evidence suggests that oxytocin administration may lead to different outcomes based on the gender [59] and sexual orientation [60] of recipients. To control for this, we limited study participation to men who have sex with men (MSM). Fifty MSM who meet the criteria for MUD will be enrolled in the current study. Participants will be recruited from community mental health programs specializing in this clinical population in San Francisco, CA, USA, including the San Francisco AIDS Foundation, the Alliance Health Project, the Positive Health Program HIV/AIDS Clinic, as well as from study flyers posted in the community. Due to the specificity of our patient population, we acknowledge that participants may recognize each other or even belong to the same social circles. While we do not formally measure pre-existing relationships among those assigned to the same therapy cohort, we do take steps to ensure that intimate partners are not assigned to the same therapy cohort by collecting names of intimate partners as well as any community member that each potential participant would *not* want to be enrolled with in group therapy.

### Inclusion and exclusion criteria

The inclusion criteria are: (1) 18 to 65 years old, (2) male-identified, (3) history of sexual contact with men, (4) meet *Diagnostic and Statistical Manual of Mental Disorders, fifth edition* (DSM-5) criteria for severe MUD, and (6) considering initiating treatment for MUD or initiated treatment within the past month. Excluded from this study are individuals with: (1) a diagnosis of bipolar I disorder, (2) evidence of opioid use within the past month, (3) severe alcohol use disorder with withdrawal symptoms, (4) suicidal ideation with intent or plan or homicidal ideation within the past 90 days, (5) suicide attempt in the past 6 months, (6) cognitive impairment or behavioral issues precluding participation in group therapy, (7) diseases likely to influence hormonal or neuroendocrine status or taking hormone supplements, (8) conditions preventing nasal spray administration (e.g., nasal obstruction or frequent nose bleeds), and (7) allergies to E216, E218, or chlorobutanol hemihydrate (preservatives in nasal spray).

### Identification of target sample (eligibility screen)

**Fig. 1:**
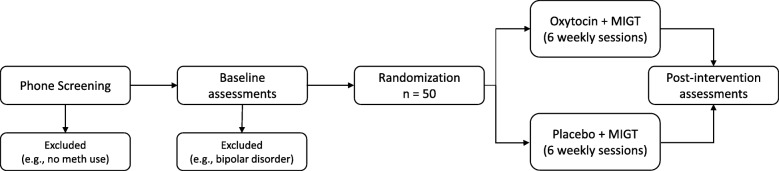
Study flow diagram

Interested individuals will undergo initial screening for eligibility using a brief, structured telephone interview. To determine full eligibility, participants will attend an initial in-person assessment where informed written consent will be obtained by study staff prior to any study procedures. See Fig. 1 for study flow diagram. Sociodemographic variables will be collected by a clinical assessor, including: age, gender identification, sexual orientation using the Kinsey Scale [61], race, education, employment status, annual income, living situation, relationship status, HIV status, medical history, and medications. The subsections of Major Depressive Disorder, Manic Episodes, Alcohol Use Disorder, Substance Use Disorders, and Psychotic Disorders of The Mini International Neuropsychiatric Interview (MINI) 7.0.0 [62] and the Suicide subsection from the Structured Clinical Interview for DSM-5 (SCID-5) [63] will be administered. Participants will provide a urine sample and undergo additional clinical interviews and self-assessment questionnaires at this initial visit. A study clinician will perform a brief medical examination, including examination of the nasal mucosa.

### Procedure

Eligible individuals enrolled in the study will be randomized, using a computer-generated random number sequence, to receive MIGT along with either oxytocin or placebo administration prior to each session. Each member of a specific MIGT cohort will receive the same study drug as the other members of that cohort, and members of each MIGT cohort will receive the same study drug for all six sessions. Blinding procedures will be controlled by Wellspring Compounding Pharmacy (Berkeley, CA, USA).

Previous work has shown that intranasal oxytocin administration begins to have physiological effects within 30 min and lasts for at least 90 min [35]. Thirty minutes prior to beginning each MIGT session, the study physician will administer the study drug (Wellspring Compounding Pharmacy, Berkeley, CA, USA) intranasally using a mucosal atomization device (MAD300; Teleflex technologies, Mooresville, NC, USA) attached to a 1-mL Luer-lock syringe. The oxytocin will be prepared as 40 International Units (IU) of oxytocin per 1 mL. Participants will receive 1 mL total, with 0.5 mL administered in each nostril using two separate syringes (with 0.06 mL additional drug in the first syringe to account for the MAD300 dead space). The administration procedures are consistent with recommendations for standardized intranasal drug administration [64]. Study drug administration will occur with all members present in the group therapy space, and the order of administration to individuals within a cohort will be randomized at each session.

To test tolerability to the study drug, participants will complete an Oxytocin Side Effect Checklist (OTSE), a list of 21 possible side effects [37] and the opportunity to report any other adverse effects, after each MIGT session. In addition, participants will be asked after each session to guess whether they have received oxytocin or placebo.

#### Substance use data collection

A urine sample will be collected at each study visit. A point-of-care, Clinical Laboratory Improvement Amendments (CLIA)-waived, 10-panel, Toxicology iCup Dx drug screen (Alere Inc., Waltham, MA, USA) will be used to screen samples for methamphetamine and other common substances of abuse.

#### Audiovisual data collection

Audio and video of each 90-min MIGT session will be recorded. The audio recording will be captured using the Blue Yeti USB omnidirectional microphone (Bluemic, Westlake Village, CA, USA) and recorded using Audacity audio software (Version 2.1.3, 2017) at a sample rate of 48,000 Hz. Each co-facilitator and group member will be video recorded individually using a Google Nexus 7 Tablet (Google, Menlo Park, CA, USA) equipped with a 5.0-MP camera and recorded at a 1280 × 720 resolution and 30 frames per second.

#### Psychophysiology data collection

Participants and co-facilitators will each be equipped with a Zephyr™ BioHarness v3.0 (Zephyr Technology, Auckland, New Zealand), wearable, wireless, psychophysiology data collection equipment. Once a clear signal is established, participants will be comfortable seated for a silent, 5-min baseline physiology recording. The Zephyr™ TEAM System using OmniSense Software (Version 4.2.4) will record a continuous electrocardiogram (sampled at 250 Hz), respiratory rate, temperature, and three-axis accelerometry to measure posture and activity level throughout each 90-min MIGT session.

Psychophysiology data will be pre-processed using MindWare HRV software (v3.1.2, MindWare Technologies, Ltd., Gahanna, OH, USA). Artifacts will be identified via MindWare’s dual MAD/MED and IBI Min/Max artifact detection algorithms derived from established strategy [65] and then processed manually. High-frequency heart rate variability (HF-HRV) is used as an indicator of parasympathetic nervous system activity and a primary psychophysiological variable of interest with regard to human social behavior. HRV is reduced in methamphetamine users when compared to matched healthy controls [66], and oxytocin has been previously shown to increase HRV via variable changes of increased HF-HRV and decreased Detrended Fluctuation Analysis (DFA-1), which measures how “self-similar” the data are [67]. Evidence suggests that autonomic cardiac control may moderate the relationship between oxytocin and social behavior, and HRV may play a key role in psychiatric patient response in treatment trials of oxytocin [68].

#### Self-assessment measures

**Fig. 2:**
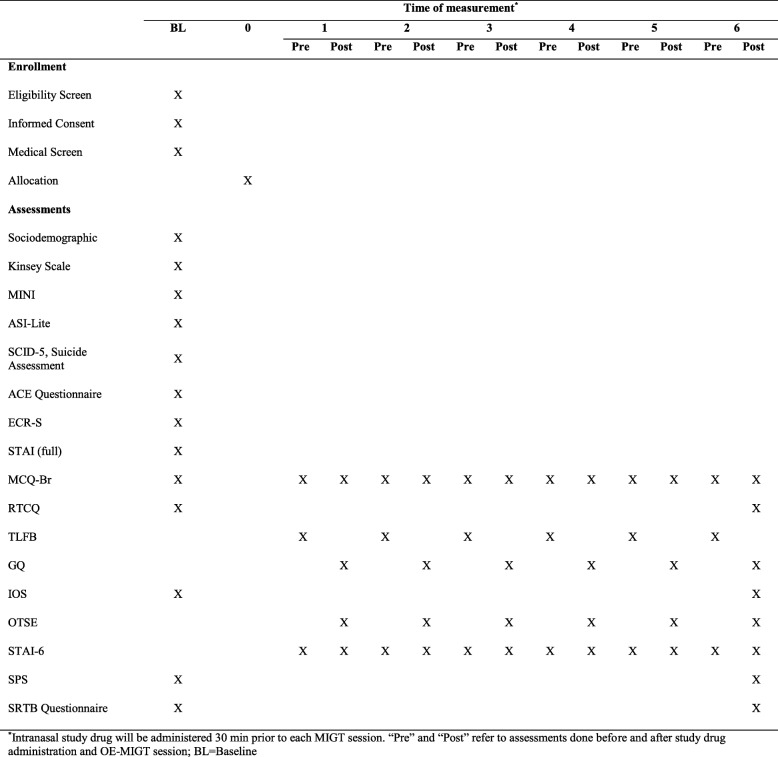
Eligibility and assessment timetable

Participants will complete self-assessment measures on Google Nexus 7 Tablets via Research Electronic Data Capture (REDCap), an online data capture tool, hosted by the University of California, San Francisco. At the initial visit, participants will be asked their preferred term for methamphetamine, and all future references to the drug in self-assessment questionnaires will use the individual respondent’s preferred term. The timing of administration of self-assessment measures is shown in Fig. 2.

1. *Social and group cohesion measures*
  1.1 The Group Questionnaire (GQ) [69], a 30-item measure of the quality of the therapeutic relationships in a group setting
  1.2 The Social Provisions Scale (SPS) [70], a 24-item measure of the degree to which respondents’ social relationships provide various dimensions of support
  1.3 The Inclusion of Others in the Self (IOS) [71], a measure assessing perceived interpersonal connectedness to various “others” for which respondents choose one in a series of images of increasingly overlapping circles. The current study measures participants’ relationships with: methamphetamine, methamphetamine-using communities, the LBGTQ community, their most intimate relationship, and their family of origin. The final administration of this questionnaire also asks about their relationship with their MIGT cohort for the study
  1.4 The Sexual Risk-Taking Behavior (SRTB) questionnaire [9, 72] will be used to conduct a structured interview to assess risky sexual behaviors over the past 6 weeks. This measure asks the same questions regarding sex while sober and sex while under the influence of methamphetamine
2. *Stress reactivity measures*
  2.1 The Six-Item State-Trait Anxiety Inventory Short Form (STAI-6) [73], a validated measure of the three highest anxiety-present and three highest anxiety-absent items from the state portion of the full State-Trait Anxiety Inventory [74]
3. *Substance use measures*
  3.1 The Methamphetamine Craving Questionnaire-Brief (MCQ-Br), adapted from the Stimulant Craving Questionnaire-Brief [75], a 10-item measure of methamphetamine craving using a 7-point Likert scale
  3.2 Self-reported methamphetamine use as measured by a Timeline Follow Back (TLFB), self-administered in our protocol [76], an assessment of daily use estimates for alcohol and other substances over the past 7 days [77]
  3.3 The Readiness to Change Questionnaire (RTCQ) [78], adapted for methamphetamine users, is a 12-item measure based on the stages-of-change model [79]

Psychometric variables, including sociodemographic and diagnostic measures described above, that are pertinent to the unique combination of MUD, MIGT, and oxytocin administration, will be collected during the initial screening visit. Additional variables will include the following: The Experiences in Close Relationship Scale – Short Form (ECR-S) [80], which is a 12-item measure of the construct of adult attachment, resulting in scores for attachment anxiety and attachment avoidance. For this study, we will substitute the term “romantic” for “intimate,” which we defined as “emotional closeness, including close family relationships and/or romantic relationships.” The Adverse Childhood Experiences (ACE) Questionnaire [81] is a 10-item measure of childhood experiences of abuse and neglect. ACE scores have a strong graded relationship to the risk of developing and maintaining substance use disorders [82] and modify the relationship between intranasally administered oxytocin and cortisol reactivity in stimulant users [31]. The State-Trait Anxiety Inventory (STAI) [74] is a 40-item measure; 20 items assess for state anxiety and 20 items assess for trait anxiety. Lastly, subsections of the Addiction Severity Index-Lite (ASI-Lite) [83] will be used to assess quantity of substance use in the past 30 days as well as lifetime use patterns.

#### Data management

All electronic data will be stored on encrypted hard drives or password-protected computers. Hard copies of any data will be kept in locked filing cabinets in a secure office. All questionnaire data will be de-identified. An independent data monitoring committee (DMC) will review accumulated data on a regular basis. Additionally, the study’s Institutional Review Board (IRB) will monitor the trial and conduct an audit once per year.

### Data Analysis Plan

Descriptive statistics will be used for demographic information and other baseline characteristics. Our dichotomous primary outcome measure (in attendance/absent) will be compared between oxytocin and placebo treatment conditions using generalized estimating equations to accommodate for correlations among the observations originating from the same participant and correct for interdependence resulting from MIGT cohort membership. Secondary outcomes collected at each MIGT session consist of group cohesion as measured by the GQ, self-reported anxiety as measured by the STAI-6, psychophysiological measures (including heart rate, HF-HRV, and temperature), methamphetamine craving as measured by the MCQ-Br, and urine drug screen. Given the exploratory nature of this novel study design, we will use descriptive statistics and estimate Cohen’s *d* effect sizes for secondary outcomes. Effect sizes will be calculated by subtracting the mean change in the oxytocin group from the mean change in the placebo group and dividing by the pooled baseline standard deviation. If there is substantial missing data, we also will consider using mixed linear models or multiple-imputation techniques to estimate mean change values in each treatment group with greater accuracy. Additional exploratory outcome measures collected at two timepoints, baseline and after the final MIGT session, consist of the SRTB, SPS, IOS, and RTCQ and will be processed using the same methodology. Results of secondary outcomes will be considered exploratory, and further confirmatory studies would be needed to confirm the results. ECR-S, ACE, STAI, and subsections of the ASI-Lite, along with baseline measurements of the SPS, IOS, SRTB, and RTCQ, will be explored as potential predictors of oxytocin response using Pearson’s correlation coefficients.

In addition to utilizing psychophysiology measures recorded during MIGT sessions as a secondary outcome variable in our main data analysis approach, we will utilize models designed to explore physiological synchrony, contagion, and other aspects of shared physiological states [84].

## Discussion

The high prevalence of MUD among MSM leads to significant health disparities, including increased risk for HIV seroconversion. Currently, no psychopharmacological agent is FDA-approved for the treatment of MUD. This investigation represents the first randomized clinical trial to administer the social peptide, oxytocin, to individuals with MUD. Data from this study should inform further development of oxytocin’s role in psychopharmacological-psychosocial combination treatment. Outcomes aim to further elucidate the potential mechanisms of OE-MIGT for MUD. Overall, this study aims to address methamphetamine use, treatment engagement, and stress reactivity in a difficult-to-treat population for whom current attrition rates are high (Additional file 1).

### Limitations and future directions

The current study will only include men who identify as having sex with men; therefore, results may not generalize to other genders and sexual orientations. There may be further selection bias regarding the motivation and engagement of participants who choose to take part in our study protocol. Furthermore, studies with substance use disorder populations often must deal with missing data from attrition. Our self-report measures increase the risk of reporting bias, although objective measures of psychophysiology will also be examined. Lastly, group therapy studies present inherent complications regarding statistical analyses. We will take these factors into account, although likely at the expense of statistical power. Despite these limitations, this study has many novel features and effect sizes will contribute to ongoing research. Future goals involve the further development of oxytocin-enhanced psychotherapy and expanding this treatment modality to other patient populations.

## Trial status

Protocol version 1.0. Recruitment started on 27 March 2017 and is estimated to end around 31 December 2018.

## Additional file

Additional file 1: Standard Protocol Items: Recommendations for Interventional Trials (SPIRIT) 2013 Checklist: recommended items to address in a clinical trial protocol and related documents^*^. (DOC 121 kb)

## Abbreviations

ACE: Adverse Childhood Experiences
AIDS: Acquired immune deficiency syndrome
ANOVA: Analysis of variance
ASI-Lite: Addiction Severity Index-Lite
CLIA: Clinical Laboratory Improvement Amendments
DFA-1: Detrended Fluctuation Analysis
ECR-S: Experiences in Close Relationships-Short
GQ: Group Questionnaire
HF-HRV: High-frequency heart rate variability
HIV: Human immunodeficiency virus
HRV: Heart rate variability
IOS: Inclusion of Others in the Self
MCQ-Br: Meth Craving Questionnaire-Brief
MI: Motivational interviewing
MIGT: Motivational interviewing group therapy
MINI: Mini International Neuropsychiatric Interview
MITI: Motivational Interviewing Treatment Integrity
MSM: Men who have sex with men
MUD: Methamphetamine use disorder
OE-MIGT: Oxytocin-enhanced motivational interviewing group therapy
OTSE: Oxytocin Side Effect Checklist
RTCQ: Readiness to Change Questionnaire
SCID-5: Structured Clinical Interview for DSM-5
SPS: Social Provisions Scale
SRTB: Sexual Risk-Taking Behavior questionnaire
STAI: State-Trait Anxiety Inventory
STAI-6: Six-item State-Trait Anxiety Inventory
TLFB: Timeline Follow Back
US: United States
